# The Use of Inhaled Epoprostenol for Acute Respiratory Distress Syndrome Secondary Due To COVID-19: a Case Series

**DOI:** 10.2478/jccm-2021-0037

**Published:** 2021-11-17

**Authors:** Komal Imtiaz, Wade Jodeh, Dave Sudekum, Bruno DiGiovine, Jason Hecht

**Affiliations:** 1 University of Texas at Houston, Houston, TX, USA; 2 St Joseph Mercy Ann Arbor. Ann Arbor, MI, USA

**Keywords:** epoprostenol, acute respiratory distress syndrome, severe acute respiratory syndrome, coronavirus

## Abstract

**Introduction:**

Inhaled epoprostenol (iEpo) is a pulmonary vasodilator used to treat refractory respiratory failure, including that caused by Coronavirus 2019 (COVID-19) pneumonia.

**Aim of Study:**

To describe the experience at three teaching hospitals using iEpo for severe respiratory failure due to COVID-19 and evaluate its efficacy in improving oxygenation.

**Methods:**

Fifteen patients were included who received iEpo, had confirmed COVID-19 and had an arterial blood gas measurement in the 12 hours before and 24 hours after iEpo initiation.

**Results:**

Eleven patients received prone ventilation before iEpo (73.3%), and six (40%) were paralyzed. The partial pressure of arterial oxygen to fraction of inspired oxygen (P/F ratio) improved from 95.7 mmHg to 118.9 mmHg (p=0.279) following iEpo initiation. In the nine patients with severe ARDS, the mean P/F ratio improved from 66.1 mmHg to 95.7 mmHg (p=0.317). Ultimately, four patients (26.7%) were extubated after an average of 9.9 days post-initiation.

**Conclusions:**

The findings demonstrated a trend towards improvement in oxygenation in critically ill COVID-19 patients. Although limited by the small sample size, the results of this case series portend further investigation into the role of iEpo for severe respiratory failure associated with COVID-19.

## Introduction

Coronavirus disease 2019 (COVID-19) has emerged as a global pandemic, from the first case described in Wuhan, China, in December 2019, to almost 7 million cases and over 400,000 deaths worldwide as of June 2020.^1^ Recent data suggest the rate of critical illness lies between 5-22%, with corresponding mortality up to 61% in the critically ill [1, 2, 3, 4]. The rapid progression to respiratory failure and development of acute respiratory distress syndrome (ARDS) remain the most salient features in critically ill patients [5]. Rates of invasive mechanical ventilation are nearly 80% in this select group. The partial pressure of arterial oxygen to the fraction of inspired oxygen (P/F ratio) is consistent with moderate to severe ARDS [3, 4].

ARDS is a life-threatening syndrome with clinical manifestations of severe hypoxemia, intense lung inflammation, abnormal pulmonary compliance, diffuse alveolar injury, and extensive epithelial destruction [6, 7]. The cornerstone of ARDS treatment is lung-protective ventilation with low tidal volumes, in conjunction with conservative fluid management and higher positive end-expiratory pressure (PEEP). [8, 9, 10, 11]. For cases of refractory hypoxemic respiratory failure in ARDS, salvage therapies include prone positioning, neuromuscular blockade (NMB), inhaled pulmonary vasodilators and extracorporeal membrane oxygenation (ECMO) [7]. The Surviving Sepsis Campaign provides recommendations for the management of ARDS in COVID-19. However, with limited published clinical knowledge, these recommendations are mostly weak with low-quality evidence [12].

The rapid development of respiratory failure in COVID-19, without any definitive antiviral therapies, and limited access to ECMO centres, has forced many clinicians into using inhaled pulmonary vasodilators as therapies of last resort.

Historically, the pulmonary vasodilators, nitric oxide (iNO) and epoprostenol (iEpo) have been shown to improve oxygenation in ARDS with no effect on mortality, ventilator-free days, or attenuation in disease severity [13, 14].

The popularity of iEpo is growing due to its similar efficacy, lower cost and reduced toxicities compared to iNO [6, 7, 15, 16]. Despite improving oxygenation in nearly 60% of patients with ARDS, iNO has not been shown to improve outcomes generally [16, 17].

iEpo’s half-life is 3-4 minutes, and its effects resolve within twenty-four minutes after discontinuation of use [18, 19]. Furthermore, there have been no drug interactions of major clinical significance identified [19, 20].

In addition to its potent vasodilatory effect, iEpo has anti-inflammatory and antiplatelet aggregation properties, which may provide a mechanistic benefit in ARDS and COVID-19 [6, 13].

iEpo has also been implicated as a potential inhibitor of severe acute respiratory syndrome coronavirus 2 (SARS-CoV-2) replication [21, 22]. In early 2020, Farag et al. (2020) used a structure-based drug repositioning strategy to assess hundreds of drugs that could target a pro-substrate-binding pocket of SARS-CoV-2. iEpo was amongst the agents listed to have potential clinical utility in this respect, though it was concluded that this required further biological validation [23]. At a biochemical level, epoprostenol forms four hydrogen bonds with SARS-CoV-2 M^pro^ via in silico predictions; its substrate specificity profiling of SARS-CoV-2 M^pro^ protease provides the basis for anti-COVID-19 drug design, which warrants further investigation [21].

With the critical nature of this disease and the lack of evidence-based therapies, we hoped to contribute to the ever-growing body of literature needed by clinicians. Unfortunately, no published studies describing the use of inhaled pulmonary vasodilators in treating ARDS in COVID-19 patients were found in the literature current at the time of writing.

The cases series aims to describe the experience of treating a cohort of critically ill COVID-19 patients with inhaled epoprostenol in three institutions.

## Methods

This retrospective case series took place from April 1st 2020, to May 31st 2020, on consecutive patients admitted to three hospitals in southeast Michigan that are part of St. Joseph Mercy Health System. This case series was approved by the St. Joseph Mercy Ann Arbor Institutional Review Board and granted exempt status. St. Joseph Mercy Ann Arbor and St. Joseph Mercy Oakland are teaching hospitals and tertiary care centres. In contrast, St. Joseph Mercy Livonia is a teaching hospital with adult intensive care units near the previously mentioned referral centres.

Patients were included in the analysis if they received iEpo and had a laboratory-confirmed COVID-19 infection via a reverse-transcriptase–polymerase-chain-reaction assay of a specimen collected on a nasopharyngeal swab.

Patients were excluded if they did not have a documented arterial blood gas (ABG) in the previous 12 hours before initiating iEpo, were < 18 years of age, or were pregnant. Patients were also excluded if they did not have an ABG within 24 hours after starting iEpo for a reason other than death (e.g. transfer to another hospital).

Patient records of those who received iEpo were obtained via reporting functions available within the electronic medical record. Data collection on all patient characteristics was completed manually by study investigators, with an independent double-check completed on each patient to ensure accuracy. Pertinent comorbidities were collected, including immunocom-promised status as defined by viral immunosuppression, neoplastic disease, immunosuppressive drugs, chemotherapy, active hematologic malignancy, or active neoplasm. Ventilator settings and ABGs were collected from the closest documented information before initiation of iEpo. All subsequent ABGs were collected following the initiation of iEpo.

Data regarding COVID-19 specific treatment therapies, including hydroxychloroquine, tocilizumab, convalescent plasma, remdesivir, and corticosteroids, was obtained.

The “use of corticosteroids” was defined as any new start of corticosteroid treatment for ARDS or COVID-19 based on the discretion of study investigators after reviewing the medical record.

At the three named facilities, iEpo is approved for use only in the ICU under the care of an intensivist. There is no set protocols or criteria at any of the institutions for patient selection before initiating iEpo. Therefore, the decision to initiate therapy was provider dependent.

A dedicated pump and Aerogen Solo Nebulizer System (Aerogen, Galway, Ireland) was used for iEpo administration, and the ICU nurses and respiratory therapists closely monitor all patients.

Monitoring parameters include vital signs, arterial blood gases (PaO_2_, P/F ratio), central venous oxygen saturation and cardiac index or pulmonary artery pressures (only in patients with pulmonary catheters). Dosing protocols varied by institution.

One institution utilizes 30,000 nanogram/mL concentrations of continuous nebulization at a flow rate of 50 ng/kg/ml with subsequent weans decreasing by 10ng/kg/min. The other institutions utilize a starting concentration of 20,000 nanograms/mL with a fixed continuous flow rate of 9 mL/hr and weans to the concentration by 50% based on clinical improvement.

Descriptive outcomes of interest included change in P/F ratio following initiation of iEpo, clinical improvement defined as a 10% increase in P/F ratio, hospital mortality, time to first wean of mechanical ventilation defined as a decrease in FiO2 or PEEP from before initiation, time to extubation, and days of iEpo therapy.

Definitions of ARDS were derived from the Berlin Criteria, with severe ARDS having a P/F ratio < 100 mmHg (AP). Descriptive statistics and paired t-tests were performed utilizing Microsoft Excel 2016 (Redmond, Washington), with a defined significance level set at ***α*** = 0.05.

## Results

### Characteristics of Patients Before iEpo Administration

**Fig. 1:**
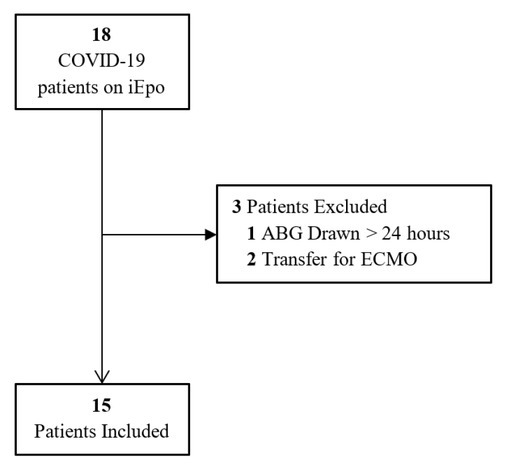
Study Population Abbreviations: COVID-19, coronavirus disease 2019; iEpo, inhaled epoprostenol; ECMO, extracorporeal membrance oxygenation; ABG, arterial blood gas

During the defined study period, 18 critically ill patients with COVID-19 managed with iEpo were identified, with 15 ultimately meeting inclusion criteria (Figure 1).

The baseline characteristics before receiving iEpo are shown in Table 1. The mean (SD) age of the patients was 57.2(13.7). 73.3% of patients were male, 26.7 % were female.

**Table 1:** Baseline Characteristics Prior to Initiation

	Epoprostenol (N=15)
Age, years ± SD	57.2 ± 13.7

Male, No (%)	11 (73.3)

BMI, kg/m^2^ ± SD	33.0 ± 10.9

SOFA score, ± SD	8.9 ± 3.3

COVID-19 Treatments	
Hydroxychloroquine	12 (80.0)
Tocilizumab	4 (26.7)
Corticosteroids	13 (86.7)
Convalescent Plasma	3 (20.0)
Remdesevir	5 (33.3)

Vasopressor, No (%)	13 (86.7)

Ventilator Status, No (%)	
VC/AC	4 (26.7)
PC/AC	10 (66.7)
APRV	1 (6.7)
Prone	11 (73.3)
Paralyzed	6 (40.0)
Arterial Blood Gas	
FiO_2_, % ± SD	91.3 ± 13.4
PEEP, cm H_2_0 ± SD	13.7 ± 3.3
pH, ± SD	7.33 ± 0.1
pCO_2_, mmHg ± SD	48.8 ± 12.4
Pa0_2_, mmHg ± SD	84.7 ± 33.2
P/F Ratio, mmHg ± SD	95.9 ± 42.0

Comorbidities, No (%)	
Asthma	2 (13.3)
COPD	0 (0.0)
CAD	4 (26.7)
Diabetes	6 (40.0)
Immunocompromised	3 (20.0)

Abbreviations: SD, standard deviation; BMI, body mass index; SOFA, sequential organ failure assessment; COVID-19, Coronavirus Disease 2019; VC/AC, volume control assist control; PC/AC, pressure control assist control; APRV, airway pressure release ventilation; Fi02, fraction of inspired oxygen; PEEP, positive end expiratory pressure; pCO_2_; partial pressure of carbon dioxide; mmHg, millimeters of mercury; Pa02, partial pressure of oxygen; P/F, Pa02/Fi02; COPD, chronic obstructive pulmonary disease; CAD, coronary artery disease

The patients’ mean BMI was 33(10.9) kg/m^2^; The mean SOFA Score was 8.9(3.3); and 13/15 (86.7%) were on vasopressors.

At the time of administration, nine patients (60%) were receiving renal replacement therapy; in eight of these, this was due to end-stage renal disease and one to acute kidney injury.

Therapies directed against COVID-19 were tracked. Twelve patients (80%) were treated with hydroxychloroquine (Zydus Pharmaceuticals, Pennington, New Jersey), four (26.7%), with tocilizumab (Genentech, INC, San Francisco, California), 13 (86.7%) received glucocorticoids, five (33.3%) received remdesivir (Gilead Sciences, Foster City, California), and three (20%) had received convalescent plasma.

All patients were on mechanical ventilation before receiving iEpo. Eleven patients were receiving prone ventilation before iEpo (73.3%), and six patients (40%) were chemically paralyzed with cisatracurium (Meitheal Pharmaceuticals, Chicago, Illinois), rocuronium (Auromedics Pharma, East Windsor, New Jersey), or vecuronium (Auromedics Pharma East Windsor, New Jersey).

The last ABG before iEpo initiation showed a mean fraction of inhaled oxygen (FiO_2_) of 91.3% and a P/F ratio of 95.9 mmHg.

### Clinical Data following iEpo Administration

Clinical outcomes were recorded following iEpo initiation and are reported for each patient in Table 2.

**Table 2:** Clinical Data for Fifteen Inhaled Epoprostenol Patients

	**Patient**	**1**	**2**	**3**	**4**	**5**	**6**	**7**	**8**	**9**	**10**	**11**	**12**	**13**	**14**	**15**	**Summary**
**Prior to Initiation**	Prone	No	No	Yes	Yes	No	Yes	Yes	Yes	Yes	No	Yes	Yes	Yes	Yes	Yes	11 (73%)
	Paralyzed	No	No	No	No	No	Yes	Yes	No	No	Yes	Yes	Yes	No	No	Yes	6 (40%)
	Mode	PC/AC	PC/AC	PC/AC	VC/AC	VC/AC	APRV	PC/AC	PC/AC	PC/AC	VC/AC	VC/AC	PC/AC	PC/AC	PC/AC	PC/AC	^-^
	FiO_2_ (%)	75	100	90	70	60	100	100	100	80	100	100	95	100	100	100	91.3
	PEEP (cm H_2_O)	18	12	15	20	10	12	12	14	14	10	12	12	15	10	20	13.7
	PaO_2_ (mmHg)	119	89	53	92	68	141	48	72	136	46	89	68	43	129	77	84.7
	P/F (mmHg)	159	89	59	131	113	141	48	72	170	46	89	72	43	129	77	95.9

**Following Initiation**	Prone	No	No	Yes	No	No	Deceased prior to additional labs	No	Yes	Yes	No	No	Yes	Yes	Yes	Yes	7 (47%)
	Paralyzed	No	No	No	No	No		No	No	No	No	No	Yes	Yes	No	Yes	3 (20%)
	Mode	VC/AC	PC/AC	APRV	PC/AC	VC/AC		APRV	PC/AC	PC/AC	VC/AC	VC/AC	PC/AC	PC/AC	PC/AC	PC/AC	-
	FiO _2_ (%)	50	100	100	70	80		100	80	70	100	40	95	95	85	100	83.2
	PEEP (cm H_2_O)	12	14	30	20	10		13	14	16	10	12	12	15	10	24	15.1
	PaO_2_ (mmHg)	99	212	65	201	62		60	55	89	64	52	73	73	97	108	93.6
	P/F (mmHg)	198	212	65	287	78		60	69	127	64	130	77	77	114	108	119.0
	Time Wean to (hours) first MV	8	2	18.5	12.1	2.3	N/A	13	0.2	6	N/A	4.4		3	5.3	N/A	6.8
	Time to Extubation (days)	9.2	11.0	8.7	N/A	N/A	N/A	N/A	N/A	N/A	10.8	N/A	N/A*	N/A		N/A	9.9
	Days of Therapy	5	3	3	3	7	1	2	5	3	2	1		4	N/A*	2	3.1
	Mortality	No	No	No	Yes	Yes	Yes	Yes	Yes	Yes	Yes	Yes		Yes		Yes	10 (67%)

* Transferred to ECMO center therefore data unavailable Abbreviations: PC/AC, pressure control assist control; VC/AC, volume control assist control; APRV, airway pressure release ventilation; FiO2, fraction of inspired oxygen; PEEP, positive end expiratory pressure; pCO2; partial pressure of carbon dioxide; mmHg, millimeters of mercury; PaO2, partial pressure of oxygen; P/F, PaO2/FiO2

**Fig. 2:**
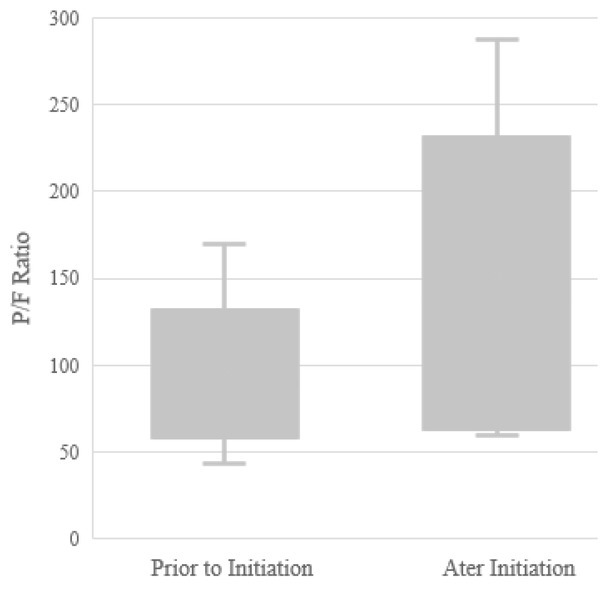
**P/F Ratio Following Initiation**. Black line represents median; X, means; box ends, interquartile ranges; capped end lines, maximum and minimum values Abbreviations: P/F, Pa02 / FiO2.

Following initiation, the first ABG occurred at an average of 204 minutes (median - 112 minutes) after starting iEpo. The mean P/F ratio was 119.0 mmHg following initiation, although not a statistically significant increase (p = 0.279) from baseline (Figure 2). The mean increase in the P/F ratio from baseline following initiation was 26.4 mmHg. Overall, ten patients (66.7%) had at least a 10% improvement in P/F ratio following initiation, indicating a positive response to therapy. On the first repeat ABG, the number of patients requiring prone ventilation decreased to 7 (46.7%), and paralysis decreased to 3 patients (20%). The second ABG following initiation, with data recorded on 13 patients, occurred at an average of 20.3 hours and showed a P/F value of 131.8 mmHg. Twelve patients had their first ABG after initiation within 5 hours, and in those cases, the P/F ratio improved from 97.2 mmHg to 127.6 mmHg (p = 0.218). When looking at the nine patients with severe ARDS before initiation, the P/F ratio improved from 66.1 mmHg to 95.7 mmHg on subsequent ABG (p = 0.317). The number of patients meeting criteria for severe ARDS decreased from 9 to 7 at the subsequent ABG. Patients who were not proned at the time of initiation had an improvement in P/F ratio following initiation of iEpo of 36.1 mmHg compared to an improvement of 26.5 mmHg in proned patients (p = 0.06).

Following the initial ABG, two patients were transferred to an outside institution for ECMO evaluation, and consequently, their data records were not complete.

Hospital outcomes, including duration of therapy, are detailed in Table 3.

**Table 3:** Hospital Outcomes

	Epoprostenol (N=12)
Hospital Mortality, no (%)	10 (66.7)
Time to first MV wean, hours ± SD	6.8 ± 5.6
Time to Extubation, days ± SD	9.9 ± 1.2
Days of Therapy, days ± SD	3.1 ± 1.6

Abbreviations: MV, mechanical ventilation; SD, standard deviation

Mortality occurred in 10 out of the 13 patients (76.9%) who had a complete data collection. Following iEpo, the average time to weaning of mechanical ventilation was 6.8 hours with a median duration of 5.3 hours. Four patients (26.7%) progressed on to extubation at an average of 9.9 days after initiation.

## Discussion

This retrospective multicentre case series describes fifteen critically ill COVID-19 patients, all of whom received iEpo while mechanically ventilated.

To our knowledge, this is the first study to characterize COVID-19 patients receiving any inhaled vasodilator and analyse their outcomes. Given their respiratory failure, all of the patients had severe COVID-19. As of May 30th 2020, the most common comorbidities of hospitalized COVID-19 patients were hypertension (over 56%), obesity (over 49%) and metabolic disease, i.e. diabetes mellitus (over 42%) [24]. A similar distribution of chronic disease was recorded in the current case series.

The chosen outcomes were reflective of oxygen delivery in severe respiratory disease. Improvement of oxygenation accelerates weaning off MV and reduces the overall duration of intubation. This simple goal is imperative, as a longer duration of MV has been strongly associated with worse overall mortality and post-hospital disposition [25].

iEpo possibly improves oxygenation on multiple fronts. It exhibits anti-inflammatory suppression, which can be beneficial given the proinflammatory state of ARDS; significant expression of interleukins 1,6, 10 and tumour necrosis factor α [26].

ARDS has been known to have a wide range of causes (secondary ARDS) and levels of gas-exchange impairment. ARDS associated with COVID-19 is no different in this regard [27]. iEpo has been shown to have more beneficial effects in adult patients with secondary ARDS than primary ARDS, as shown in a systematic review by Searcy et al. (2015) [16].

At the right dose, iEpo is effective at reducing pulmonary artery pressure, thereby improving PaO_2_. Its antiplatelet effects combat the thrombo-occlusive state of ARDS, further improving oxygenation [26, 28].

Currently, iEpo is not a standard in the initial treatment of ARDS but is more often used as salvage therapy for refractory hypoxemia [8]. Upon receiving iEpo, the most overt improvement in parameters was reflected in patient oxygen delivery. In the current patient series, all but one patient demonstrated an increase in P/F ratio post initiation of iEpo. The P/F ratio defines the severity of ARDS, with more severe cases carrying higher morbidity, mortality and financial burdens [29]. The mean P/F ratio before iEpo was 95.9 mmHg reflecting severe ARDS. Within 24 hours of iEpo, the average P/F ratio improved from the “severe” range to “moderate” range (118.9 mmHg). Two patients improved to “mild ARDS” within less than 24 hours of receiving iEpo, and one patient was no longer in ARDS on the second-day post-administration.

The improvement in ARDS can be compared to the results of Tabrizi et al. (2012). A retrospective analysis of 35 patients receiving iEpo for ARDS showed that their mean P/F ratios improved from a mean of 67 to 142 at 12 hours and then to 202 at 48 hours [6].

Our patients showed a mean improvement of 37.3% in P/F ratio within 48 hours of receiving iEpo (95.9 to 131.7 mmHg). A meta-analysis assessing the use of inhaled prostaglandins in ARDS reviewed 16 studies that showed at least 39% improvement of P/F ratio and eight studies showing at least 21% improvement of PaO2 with the use of inhaled prostaglandins [13].

Similar retrospective studies have evaluated iEpo in patients with COVID-19 respiratory failure. DeGrado et al.( 2020) looked at 38 COVID-19 patients with refractory hypoxemia at a single centre who received iEpo. The median change in the P/F ratio following initiation was 0, which stands in stark contrast to the results of our analysis. However, in patients classified as responders, the median increase in the P/F ratio was 34.1 mmHg. This indicates, similar to what was found in the current series, that those select patients will positively respond to iEpo therapy with significant improvements in oxygenation [30]. Another retrospective study by Sonti et al.(2021) looked at 80 patients who received iEpo and had P/F ratios measured. Patients were initiated when they had severe ARDS, as evidenced by a P/F ratio of 92 mmHg at baseline. Similar to our analysis and that done by DeGrado, fifty per cent of patients had a clinically significant improvement in oxygenation, as evidenced by an improvement in the P/F ratio of 10%. Similar to our analysis, the most robust response to iEpo therapy was found in patients with the lowest P/F ratio upon multivariate adjustment [31].

In the crossover study by Zwissler et al. (1996), iEpo and iNO were assessed to ascertain the most effective dose for improving PaO_2_ and reducing pulmonary artery pressures.

iEpo exhibited a dose-dependent response: producing a significant increase in PaO2 of 18% at doses of 10 ng/kg/min and 24% at 25 ng/kg/min, with no effect at 1 ng/kg/min [28].

Within a single day, patients in the current series showed an average improvement in PaO2 from 84.7 to 93.5 (10.3%).

Furthermore, the lowest dose given to any patients was 10 ng/kg/min (before discontinuation), therefore never approaching the non-beneficial dose of 1 ng/kg/ min of the study by Zwissler et al.

Given the dose-dependent response demonstrated in the study, it is possible that the initiating dose of iEpo delivered to our patients carried more potential in oxygen improvement. Therefore, it would be worth investigating the optimal dosing of iEpo in ARDS secondary to COVID-19 and whether it is comparable to dosing with other causes of secondary ARDS.

In a retrospective, observational study of 52 patients with SARS-CoV-2 pneumonia admitted to an ICU in Wuhan, China, 61.5% of patients had died at 28 days. The study showed a substantial difference in the P/F ratios between survivors and non-survivors and indicated the ratio to be associated with both severity and overall prognosis. It was not documented if any patient had received any inhaled vasodilators; however, the association of prognosis and higher P/F ratios was statistically significant [2].

## Conclusion

Overall, the findings demonstrate a trend towards improvement in oxygenation of moderate-severe ARDS in COVID-19 patients through increasing values of P/F ratios, PaO2, and relatively short intervals to first wean off mechanical ventilation.

We hope that our findings portend the value of a multi-institutional prospective trial which would help confirm the efficacy of iEpo in assessing dosing strategies of iEpo delivery.
